# Fucoidan induces caspase-dependent apoptosis in MC3 human mucoepidermoid carcinoma cells

**DOI:** 10.3892/etm.2013.1368

**Published:** 2013-10-29

**Authors:** HANG-EUN LEE, EUN-SUN CHOI, JI-AE SHIN, SYNG-OOK LEE, KI-SOO PARK, NAM-PYO CHO, SUNG-DAE CHO

**Affiliations:** 1 Department of Oral Pathology, School of Dentistry, Institute of Oral Bioscience, Chonbuk National University, Jeonju 561-756, Republic of Korea; 2 Department of Veterinary Physiology and Pharmacology, Texas A&M University, College Station, TX 77843-4466, USA; 3 Department of Translational Research, Korea Health Industry Development Institute (KHIDI), Cheongwon-gun 363-951, Republic of Korea

**Keywords:** fucoidan, apoptosis, mucoepidermoid carcinoma, extracellular signal-regulated kinase 1/2, myeloid cell leukemia-1

## Abstract

Fucoidan is a sulfated polysaccharide present in brown algae that has been identified to exhibit multiple biological effects. In this study, the apoptotic effects of fucoidan in MC3 human mucoepidermoid carcinoma (MEC) cells were investigated. The apoptotic effects of fucoidan on MC3 MEC cells were evaluated by cell proliferation assay, 4′,6-diamidino-2-phenylindole staining and western blot analysis. The results showed that fucoidan decreased cell proliferation and induced caspase-dependent apoptosis in MC3 MEC cells. Fucoidan downregulated the phosphorylation of extracellular signal-regulated kinase (ERK) 1/2, whereas phospho-p38 mitogen-activated protein kinase or phospho-c-Jun NH_2_-terminal kinase (JNK) levels were not altered. In addition, fucoidan significantly decreased the expression levels of myeloid cell leukemia-1 (Mcl-1). These results suggest that fucoidan is able to modulate the ERK1/2 pathway and thereby regulate Mcl-1 protein expression and induce apoptosis in MC3 MEC cells. Therefore, fucoidan may be a promising agent for the treatment of human MEC.

## Introduction

Natural dietary compounds have been widely and safely consumed for centuries and have potential applications in pharmacology and cancer therapy (1). Fucoidan is a naturally occurring polysaccharide compound present in brown algae, including *Fucus vesiculosus*, *Cladosiphon okamuranus* and *Laminaria saccharina*(2,3). Numerous intensive studies have identified its biological activities, including antioxidative, immunomodulatory, antiviral, antithrombotic and anticoagulant effects (4–6). In addition, a number of studies support that the use of fucoidan as a supplement provides protection against various cancers (7–9). However, the anticancer effects of fucoidan in mucoepidermoid carcinoma (MEC) cells have yet to be studied.

Mitogen-activated protein kinases (MAPKs) are involved in cellular proliferation, differentiation and apoptosis (10), and the dynamic balance between extracellular signal-regulated kinase (ERK), c-Jun NH_2_-terminal kinase (JNK) and p38 MAPK contributes to the determination of cell fate (11). Previous studies have also demonstrated that MAPKs have essential roles in modulating the function of mitochondrial pro- and anti-apoptotic proteins (12,13). Myeloid cell leukemia-1 (Mcl-1), an anti-apoptotic member of the Bcl-2 family, has a pivotal role in protecting cells against apoptosis and is overexpressed in various human cancers (14). It is also important in cell survival regulatory pathways, suggesting the vital role of Mcl-1 in the regulation of apoptosis (15). Thus, MAPKs and Mcl-1 may be potential molecular targets for apoptotic cell death in cancer cells.

In the present study, the effects of fucoidan and its molecular mechanisms in the MC3 MEC cell line were investigated.

## Materials and methods

### Reagents

Fucoidan (from *Fucus vesiculosus*) and 4′,6-diamidino-2-phenylindole (DAPI) were purchased from Sigma (St. Louis, MO, USA). Dulbecco's modified Eagle's medium (DMEM), fetal bovine serum (FBS), 100× antibiotic solution, trypsin and D-phosphate-buffered saline (PBS) were obtained from WelGENE Inc. (Daegu, Republic of Korea). The poly (ADP-ribose) polymerase antibody was obtained from BD Biosciences (San Diego, CA, USA). Actin antibody was purchased from Santa Cruz Biotechnology Inc. (Santa Cruz, CA, USA). Antibodies for phospho-ERK, total ERK, phospho-JNK, total JNK, phospho-p38, total p38, Mcl-1, cleaved caspase-3 and cleaved poly ADP ribose polymerase (PARP) were purchased from Cell Signaling Technology, Inc. (Denver, MA, USA). The pan caspase inhibitor, z-VAD, was obtained from R&D Systems (Minneapolis, MN, USA).

### Cell culture and chemical treatments

MC3 MEC cells were obtained from Professor Wu Junzheng (Fourth Military Medical University, Xi'an, China). Cells were cultured in DMEM supplemented with 10% FBS and 100 U/ml each of penicillin and streptomycin in a humidified atmosphere of 5% CO_2_ at 37ºC. An equal number of cells were seeded and allowed to attach to the well plate. The cells were pretreated with a pan caspase inhibitor, z-VAD (10 μM) 1 hr before fucoidan treatment. When the cells reached 50–60% confluence, they were treated with fucoidan (25, 50 and 100 μg/ml) dissolved in 0.1% dimethyl sulfoxide (DMSO; vehicle control).

### Cell proliferation assay

Cell proliferation was determined by cell counting using a Neubauer's chamber (hemocytometer, Neubauer dual count chamber; Thermo Fisher Scientific Inc., Waltham, MA, USA). MC3 MEC cells were exposed to DMSO or fucoidan for 48 h. Following the period of exposure, cell were stained with trypan blue (0.04%) and then counted. Each experiment was carried out in triplicate and the results are expressed as the mean ± standard deviation.

### DAPI staining

The apoptotic effects of fucoidan on MC3 MEC cells were measured using a fluorescent nuclear dye, DAPI. MC3 MEC cells were seeded and treated with varied concentrations (25, 50 and 100 μg/ml) of fucoidan, harvested by trypsinization and resuspended in PBS. The cells were fixed in 100% methanol at room temperature (RT) for 10 min, deposited on slides and then stained with DAPI solution (2 mg/ml). The DAPI-stained cell morphology was observed under a fluorescence microscope (Microscope Axio Imager. M2; Carl Zeiss Co. Ltd., Seoul, Korea).

### Western blot analysis

Whole cell lysates were extracted with lysis buffer and protein concentrations were measured using a DC Protein Assay (Bio-Rad, Hercules, CA, USA). Samples containing equal concentrations of protein were separated by sodium dodecyl sulfate-polyacrylamide gel electrophoresis and then transferred to Immun-Blot™ polyvinylidene fluoride membranes (Bio-Rad). The membranes were blocked with 5% skimmed milk in Tris-buffered saline with Tween for 1 h 30 min at RT and maintained overnight at 4ºC with primary antibodies. Membranes were then incubated with horseradish peroxidase-conjugated secondary antibodies (Santa Cruz Biotechnology Inc.) at RT for 1 h 30 min. Antibody-bound proteins were detected using enhanced chemiluminescence (ECL) western blotting luminol reagent (Santa Cruz Biotechnology Inc.).

### Statistical analysis

Data were assessed for statistical significance using a Student's t-test. P<0.05 compared to that of the vehicle control was considered to indicate a statistically significant difference.

## Results

### Fucoidan inhibits cell proliferation and induces apoptosis in MC3 MEC cells

**Figure 1:**
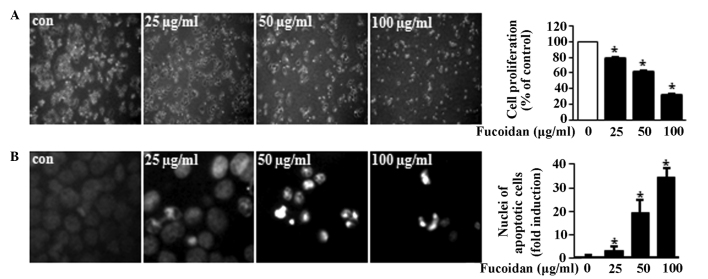
Effects of fucoidan on cell proliferation and apoptosis in MC3 MEC cells. Cells were treated with DMSO (vehicle control; con) or varied concentrations of fucoidan (25, 50, and 100 μg/ml) for 48 h. (A) Cell morphology was observed under an optical microscope and cell proliferation was determined by cell counting. The values in the bar chart represents the mean ± standard deviation (SD) of three independent experiments. ^*^P<0.05 compared with the control group. (B) Fluorescent microscope images of DAPI-stained MC3 cells. The number of cells with nuclear fragmentation and condensation were quantified and the values in the bar chart are expressed as the mean ± SD of three independent experiments. ^*^P<0.05 compared with the control group. MEC, mucoepidermoid carcinoma cancer; DMSO, dimethyl sulfoxide; DAPI, 4′,6-diamidino-2-phenylindole.

**Figure 2:**
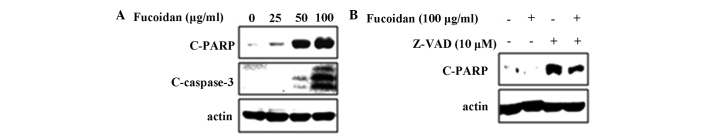
Caspase-mediated apoptosis in MC3 MEC cells. (A) Cleaved PARP and caspase 3 were detected in MC3 MEC cells treated with fucoidan by western blot analysis and actin was used as the loading control. (B) A pan caspase inhibitor (zVAD-fmk) was used to evaluate the involvement of caspase 3 in fucoidan-induced apoptosis. MEC, mucoepidermoid carcinoma cancer; PARP, poly ADP ribose polymerase.

To investigate the anticancer effects of fucoidan, the growth-inhibitory effects of fucoidan in the MC3 MEC cell line were first assessed. Cells were treated with DMSO or fucoidan (25, 50 and 100 μg/ml) for 48 h. The results demonstrated that fucoidan induced morphological changes of the MC3 MEC cells and the proliferation of the cells was significantly reduced in a concentration-dependent manner (Fig. 1A). Then, whether the growth-inhibitory effects of fucoidan were associated with apoptotic cell death was investigated. As shown in Fig. 1B, cells treated with fucoidan exhibited nuclear fragmentation and chromatin condensation in a concentration-dependent manner. The results demonstrated that fucoidan inhibited cell growth and induced apoptosis in MC3 MEC cells. The apoptotic activity of fucoidan was then determined by evaluating the levels of PARP cleavage and the activation of caspase 3. As shown in Fig. 2A, fucoidan-treated MC3 MEC cells demonstrated increased cleavage of PARP and caspase-3. To investigate the involvement of caspase 3 in fucoidan-induced apoptosis, a pan caspase inhibitor, z-VAD, was used. The results showed that the cleavage of PARP induced by fucoidan was partially blocked in the presence of z-VAD, suggesting that fucoidan-induced apoptosis is mediated by caspase activation (Fig. 2B).

### Fucoidan decreases phosphorylation of ERK1/2 but does not change phospho-p38 and phospho-JNK levels in MC3 MEC cells

**Figure 3:**
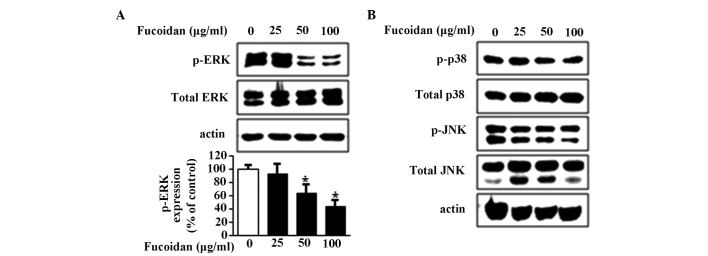
Effects of fucoidan on the activation of MAPKs (ERK1/2, p38 and JNK). MC3 MEC cells were treated with DMSO (vehicle control) or varied concentrations of fucoidan (25, 50, and 100 μg/ml) for 48 h. (A) Effects of fucoidan on phospho-ERK1/2 and ERK1/2 were detected by western blot analysis and the bar chart represents the mean ± standard deviation of three independent experiments. ^*^P<0.05 compared with the control group. (B) Phospho-p38 (p-p38), phospho-JNK (p-JNK), p38 and JNK were detected. MAPK, mitogen-activated protein kinase; ERK, extracellular signal-regulated kinase; MEC, mucoepidermoid carcinoma cancer; DMSO, dimethyl sulfoxide.

The MAPK family is positively associated with apoptotic cell death (16,17) and the MAPK signaling pathway is frequently dysregulated in neoplastic transformation (18). It has also been indicated that activation of the ERK1/2 pathway is commonly associated with survival; by contrast, the JNK1/2 and p38 MAPK pathway is associated with apoptosis (19). In the present study, the effects of fucoidan on the phosphorylation of ERK1/2, p-38 and JNK were examined, and the results showed that fucoidan downregulated the phosphorylation of ERK1/2 in a concentration-dependent manner (Fig. 3A), but did not alter the phosphorylation or total expression levels of p38 and JNK (Fig. 3B). Therefore, ERK1/2 may be important in fucoidan-induced apoptosis.

### Fucoidan downregulates Mcl-1, a downstream target of ERK1/2

**Figure 4:**
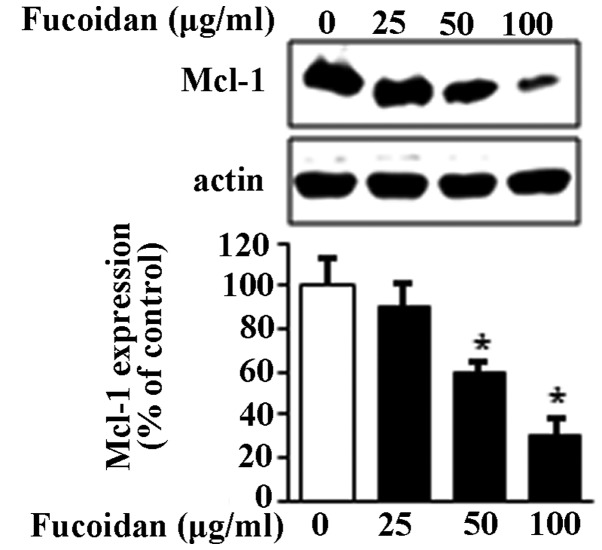
Effects of fucoidan on Mcl-1 protein expression in MC3 MEC cells. MC3 cells were treated with DMSO (vehicle control) or varied concentrations of fucoidan (25, 50, and 100 μg/ml) for 48 h and the expression of Mcl-1 protein was anlayzed by western blotting. The bar chart represents the mean ± standard deviation of three independent experiments. ^*^P<0.05 compared with the control group. MEC, mucoepidermoid carcinoma cancer; DMSO, dimethyl sulfoxide.

A number of anti-apoptotic effector proteins have been identified downstream of ERK1/2 signaling, including Mcl-1 (20,21). Thus, whether fucoidan treatment affects the Mcl-1 protein in MC3 cells was investigated using western blot analysis. The results showed that the expression of Mcl-1 protein significantly decreased with fucoidan treatment in a concentration-dependent manner (Fig. 4). These results indicated that the expression of Mcl-1 may be regulated by the ERK1/2 pathway and subsequently induce apoptosis in MC3 MEC cells.

## Discussion

Fucoidan is a potent inducer of apoptosis in various cancer cell lines (5,22). A previous study has shown that fucoidan induced extrinsic or intrinsic apoptotic signals in different cancer cell types via the altered expression or activities of mitochondria-associated proteins, cell cycle regulatory proteins, proteases and transcription factors (9). However, the molecular mechanisms by which fucoidan initiates apoptosis in MC3 MEC cells have not been characterized. In the present study, the aim was to investigate the *in vitro* anti-cancer effects of fucoidan in MC3 MEC cells. The results demonstrated that fucoidan inhibited cell growth and induced apoptosis in MC3 MEC cells, which was indicated by decreased cell proliferation, nuclear fragmentation, chromatin condensation, cleaved PARP and activated caspase 3. In addition, a pan caspase inhibitor, z-VAD, blocked fucoidan-induced apoptosis suggesting that this effect is caspase-dependent.

MAPK family members appear to be important in the regulation of cell survival (23). For example, ERK1/2 activation has been shown to promote the anti-apoptotic functions of Bcl-2 and cell survival in neuronal PC12 cells, whereas the activation of JNKs results in cell death via the apoptotic signaling pathway (24). Previous studies have identified that fucoidan induces apoptotic cell death by activating the ERK1/2 pathway (25–27); however, additional studies have also demonstrated that fucoidan is able to inactivate the ERK pathway for apoptosis (1,9,28). This suggests that the role of the ERK pathway in fucoidan-induced apoptosis remains controversial. Therefore, in the present study, the effects of fucoidan on the ERK1/2 signaling pathway were investigated. The results showed a concentration-dependent suppression of ERK1/2 phosphorylation. To eliminate the involvement of additional MAPK family members, such as JNK and p38, their expression levels were also evaluated. The results indicated that they were not altered by fucoidan, suggesting that the inactivation of ERK1/2 by fucoidan results in the induction of apoptosis.

The ERK pathway promotes cancer cell survival through inhibition of the apoptotic cascade by controlling the expression or activity of Bcl-2 family members (28,29). The fact that the Bcl-2 family and the ERK signaling pathway were both implicated in the control of cell survival suggests that ERK-stimulated enhancement of cell survival may be mediated through its effects on the expression of Bcl-2 or other Bcl-2 family members (28). Mcl-1 is an anti-apoptotic protein that is highly expressed in malignant tumors and has been implicated in resistance to chemotherapy (30). It has also been identified that ERK is an important regulator of Mcl-1 stability (31). Thus, in the present study, the effects of fucoidan on Mcl-1 were investigated, and the results showed that Mcl-1 expression was reduced by fucoidan in a concentration-dependent manner. These results suggest that fucoidan may have induced apoptosis through inactivation of the ERK pathway and the inhibition of Mcl-1.

In conclusion, to the best of our knowledge, this study demonstrated for the first time that fucoidan is able to induce apoptotic cell death in MC3 human MEC cells and this is associated with concentration-dependent inactivation of the ERK1/2 pathway to regulate Mcl-1 protein. These results suggest that fucoidan may be a promising dietary compound for the treatment of MEC.
